# CANVAS-related *RFC1* mutations in patients with immune-mediated neuropathy

**DOI:** 10.1038/s41598-023-45011-8

**Published:** 2023-10-18

**Authors:** Makito Hirano, Motoi Kuwahara, Yuko Yamagishi, Makoto Samukawa, Kanako Fujii, Shoko Yamashita, Masahiro Ando, Nobuyuki Oka, Mamoru Nagano, Taro Matsui, Toshihide Takeuchi, Kazumasa Saigoh, Susumu Kusunoki, Hiroshi Takashima, Yoshitaka Nagai

**Affiliations:** 1 Department of Neurology, Kindai University, Faculty of Medicine, Ohno-Higashi, Osakasayama, Osaka 589-8511 Japan; 2 Department of Neurology and Geriatrics, Kagoshima University Graduate School of Medical and Dental Sciences, Kagoshima, Japan; 3 Department of Neurology, NHO Minami-Kyoto Hospital, Joyo, Japan; 4 Department of Anatomy, Kindai University, Faculty of Medicine, Osakasayama, Japan; 5 Division of Neurology, Anti-Aging, and Vascular Medicine, Department of Internal Medicine, National Defense Medical College, Tokorozawa, Japan

**Keywords:** Genetics, Neuroscience, Neurology

## Abstract

Cerebellar ataxia, neuropathy, and vestibular areflexia syndrome (CANVAS) has recently been attributed to biallelic repeat expansions in *RFC1*. More recently, the disease entity has expanded to atypical phenotypes, including chronic neuropathy without cerebellar ataxia or vestibular areflexia. Very recently, *RFC1* expansions were found in patients with Sjögren syndrome who had neuropathy that did not respond to immunotherapy. In this study *RFC1* was examined in 240 patients with acute or chronic neuropathies, including 105 with Guillain-Barré syndrome or Miller Fisher syndrome, 76 with chronic inflammatory demyelinating polyneuropathy, and 59 with other types of chronic neuropathy. Biallelic *RFC1* mutations were found in three patients with immune-mediated neuropathies, including Guillain-Barré syndrome, idiopathic sensory ataxic neuropathy, or anti-myelin-associated glycoprotein (MAG) neuropathy, who responded to immunotherapies. In addition, a patient with chronic sensory autonomic neuropathy had biallelic mutations, and subclinical changes in Schwann cells on nerve biopsy. In summary, we found CANVAS-related *RFC1* mutations in patients with treatable immune-mediated neuropathy or demyelinating neuropathy.

## Introduction

Cerebellar ataxia, neuropathy, and vestibular areflexia syndrome (CANVAS) has been recently found to be caused by biallelic repeat expansions in the intron of *RFC1*^1^. More recently, the disease entity has expanded to chronic neuropathy without cerebellar ataxia or vestibular areflexia^2^. Repeat configuration includes AAGGG, ACAGG, AGGGC, or combinations thereof without clear genotype and phenotype relationship^2–4^.

Immune-mediated neuropathy can be categorized into acute and chronic. Guillain-Barré syndrome (GBS) or Miller Fisher syndrome (MFS) is representative of acute immune-mediated neuropathy, whereas chronic inflammatory demyelinating polyneuropathy (CIDP) is representative of chronic immune-mediated neuropathy. Myelin-associated glycoprotein (MAG) neuropathy, originally derived from CIDP, is characterized by sensory and autonomic neuropathy with positive serum monoclonal IgM antibody against MAG^5^ and is at least partly treatable by immunotherapy including rituximab^6^. Notably, neuropathies cannot be solely attributed to the presence of antibodies. In fact, our previous study showed that among two siblings who simultaneously suffered from *Campylobacter jejuni*-associated diarrhea and subsequently tested positive for serum anti-ganglioside antibodies, only one developed GBS, suggesting the involvement of some unknown host factors^7^.

Our previous study showed that CAG repeat expansions in *ATXN2*, which causes spinocerebellar ataxia type 2 (SCA2), were found in patients with CIDP or immune-mediated neuropathy^8^. A recent review also suggested the association between repeat expansions and various diseases, including immune-mediated diseases^9^. Very recently, *RFC1* expansions were found in patients with Sjögren syndrome who had neuropathy that did not respond to immunotherapy, suggesting that neuropathy could be attributed to *RFC1* expansions^10^. Thus, a previous study suggested that unnecessary immunotherapy should be avoided in patients who have immune-mediated diseases with *RFC1* mutations^10^. However, we speculate that immunotherapy might be effective in some patients with certain unknown conditions. We therefore hypothesized that repeat expansion may be a background genetic factor for immune-mediated neuropathy. Accordingly, the current study examined *RFC1* in 240 patients with immune-mediated neuropathy, such as GBS, CIDP, and MAG neuropathy, as well as other types of neuropathies.

## Patients and methods

### Patients

This study enrolled Japanese patients and controls from the Kinki region of Japan between 2005 and 2023. *RFC1* was analyzed in 240 Japanese patients with neuropathy (146 men and 94 women; mean age, 54 ± 18 years), including 105 with GBS or MFS (62 men and 43 women, mean age, 50 ± 19 years), 76 patients with CIDP (44 men and 32 women, mean age, 56 ± 17 years), and 59 with other types of chronic neuropathy (40 men and 19 women, mean age, 61 ± 17 years), which included eight patients with MAG neuropathy, four with paraneoplastic syndrome (without MAG antibody), three with Sjögren syndrome, and three with multifocal neuropathy. Patients with known genetic causes were excluded. The clinical diagnosis was established after the first admission for diagnostic workup when DNA samples were collected. Chronic neuropathy was herein defined as weakness or sensory disturbance in two or more limbs over at least 2 months with other signs or symptoms being minimal and corresponding abnormalities in nerve conduction studies (NCS). A control group consisting of 160 apparently healthy participants (90 men and 70 women; mean age, 64 ± 17 years) was also included. The nerve biopsy specimen was analyzed as described previously^11^.

### Genetic analyses

DNA was extracted from peripheral blood using the Qiagen Kit (Qiagen, Hilden Germany). Primers used for the amplification of the short range of the repeat region in *RFC1* were as described. When no normal size band was detected, the sample was subjected to repeat-primed polymerase chain reaction (PCR) for AAGGG (pathogenic), ACAGG (pathogenic), AGGGC (pathogenic), AGAGG (possibly pathogenic), AAGGC (possibly pathogenic), AAAGG (variable penetrance), AAAAG (likely non-pathogenic), AAAGGG (likely non-pathogenic), and AAGAG (likely non-pathogenic) repeat configurations^1,2,4^. Primers used for repeat-primed PCR are described in supplemental material. All patients provided written informed consent for genetic analyses. All methods in this study were performed in accordance with the Declaration of Helsinki. The study protocol was approved by the Institutional Review Boards of Kagoshima University and Kindai University.

### Autoantibody measurement

Anti-ganglioside antibodies were examined in patients with GBS, MFS, CIDP, and IgM paraproteinemic neuropathy using GM1, GM2, GM3, GD1a, GD1b, GD3, GT1b, and GQ1b as reported previously^12,13^. Anti-GalNAc-GD1a antibody was additionally examined in patients with GBS and MFS, and anti-GT1a antibody was examined when anti-GQb1 antibody was detected^12,13^. Anti-MAG antibody and glycolipid sulfoglucuronyl paragloboside (SGPG) antibody, cross-reactive to MAG, were examined in patients with IgM paraproteinemic neuropathy as reported previously^5^. All methods in this study were performed in accordance with the Declaration of Helsinki. The study protocol was approved by the Institutional Review Boards of Kindai University.

## Results

### *RFC1* analyses

**Figure 1:**
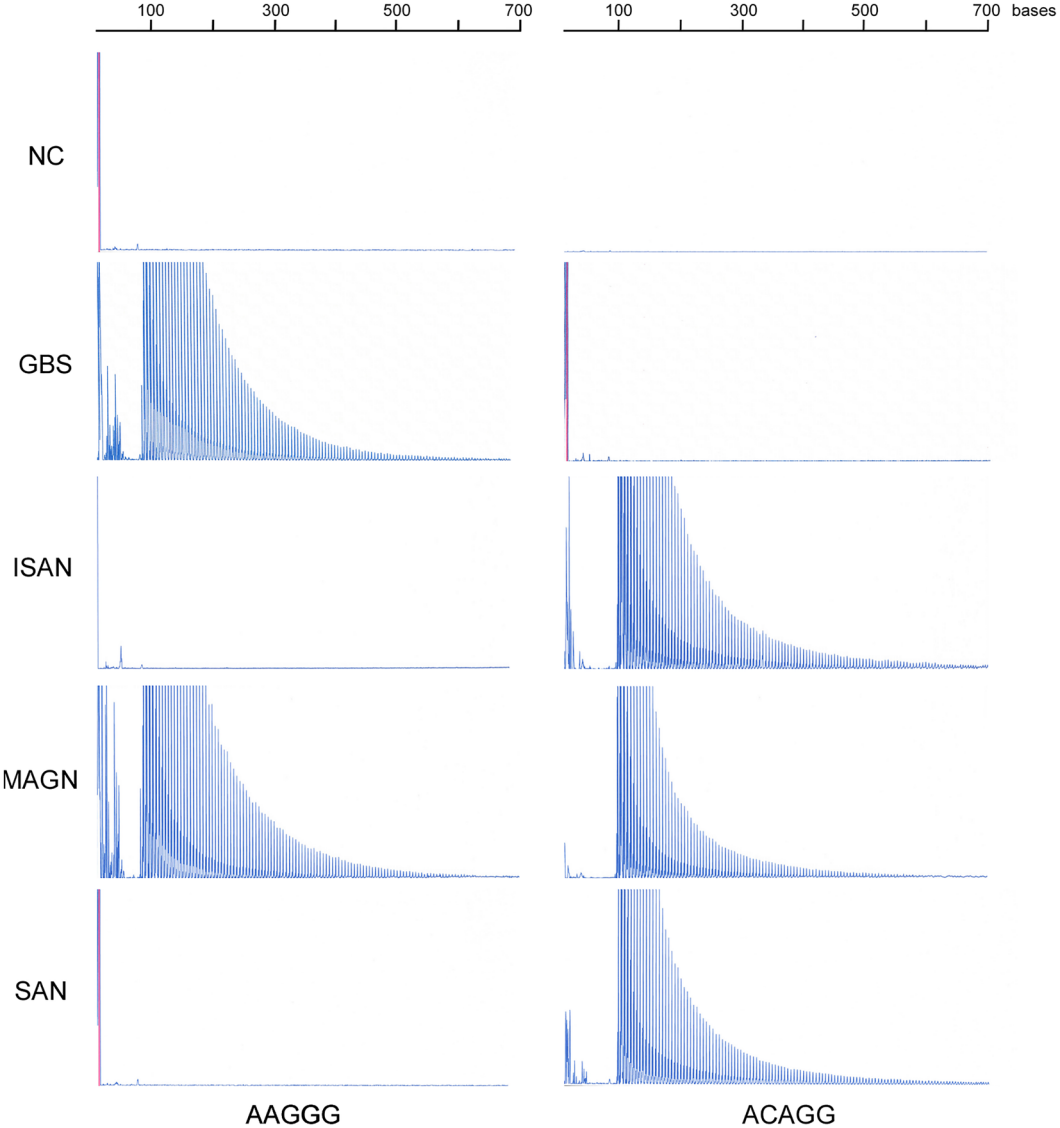
Repeat-primed PCR results for *RFC1*. Pathological repeats of AAGGG or ACAGG were expanded in patients with Guillain-Barré syndrome, idiopathic sensory ataxic neuropathy with mild motor deficit (ISAN), MAG neuropathy, or sensory autonomic neuropathy with mild motor deficit (SAN).

*RFC1* mutations were found in three patients with immune-mediated neuropathy, such as GBS, MAG neuropathy, and idiopathic sensory ataxic neuropathy and one patient with sensory autonomic neuropathy (Fig. 1). No expansion of AGGGC, AGAGG, AAGGC, AAAGG, AAAAG, AAAGGG, or AAGAG was found in the four patients. No mutations were found in the control group.

### Clinical information of patients with RFC1 mutations

Clinical and genetic information of patients with *RFC1* mutations is summarized in Table 1. Detailed clinical information is provided upon request. Results of NCS are summarized in Supplementary Table 1.

**Table 1:** Clinical and genetic information of patients with *RFC1* mutations.

Patient#	1	2	3	4
Clinical phenotype	GBS	ISAN	MAGN	SAN
Clinical course	Monophasic	Recurrent and remission	Slowly progressive	Slowly progressive
*RFC1* mutation	AAGGG biallelic	ACAGG biallelic	ACAGG/AAGGG	ACAGG biallelic
Age at recent examination	74	75	79	80
Age at onset	74	64	56	76
Sex	F	F	M	F
Autoantibody	Anti-ganglioside (IgG GQ1b, GT1a, GD1b, and GT1b)	RF (IgM anti-IgG)	IgM anti-MAG	–
Motor deficit	+ +	+	–	+
Sensory disturbances	+	+ +	+ +	+ +
Autonomic disturbances	–	–	–	+ + +
NCS	Unclassified	Axonal change + Possible demyelination at later stage	Possible demyelination	Axonal change
Sural nerve biopsy	nd	Demyelination^§^	nd	Axonal change with Schwann cell abnormalities
Treatment^§§^	1 × IVIG	8 × IVIG	12 × Rituximab (375 mg/m^2^)	2 × IVMP + 1 × IVIG
Response to immunotherapy	Good	Mild	Moderate	None
Outcome	Complete resolution of motor deficit, but mild dysesthesia remained	Subjective and objective improvement after each IVIG	Suppression of IgM levels and of symptom progression	Almost bedridden with percutaneous gastrostomy

*NCS* nerve conduction study, *GBS* Guillain Barré syndrome, *ISAN* idiopathic sensory ataxic neuropathy with mild motor deficit, *MAGN* Myelin-associated glycoprotein neuropathy, *SAN* sensory autonomic neuropathy with mild motor deficit, *RF* rheumatoid factor, –, absent; + , mild; +  + , moderate; +  +  + , severe; *nd* not done, *IVMP* intravenous methylprednisolone therapy (1 g/day for 3 days), *IVIG* intravenous immunoglobulin therapy (0.4 g/kg/day for 5 days).

^§^Data was available only from her medical record, ^§§^x means # of cycles of the treatment.

### Nerve biopsy findings of patient 4

**Figure 2:**
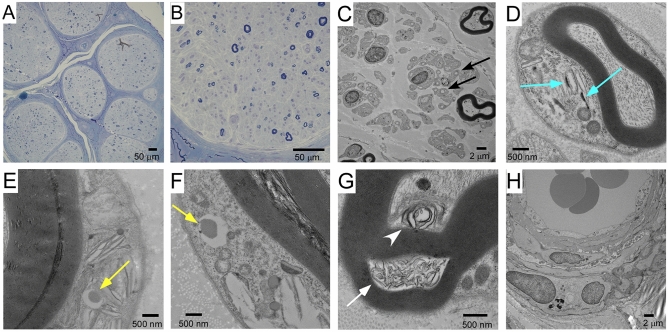
Sural nerve biopsy findings in Patient 4. (**A**) A light microscopic image showing marked loss of myelinated fibers, without amyloidosis or vasculitis. (**B**) A lager image showing marked loss of both large and small myelinated fibers. (**C**) An electron microscopic image showing many collagen pockets (black arrow), reflecting unmyelinated fiber damage. (**D**) A Schwann cell had cytoplasmic dense materials (blue arrow). (**E**) Another Schwann cell had a cytoplasmic inclusion (yellow arrow). (**F**) The other Schwann cell had a cytoplasmic inclusion similar to the one shown in E (yellow arrow). (**G**) Membranous materials were seen between myelin sheaths (white arrow) or between a myelin sheath and an axon (white arrowhead). (**H**) Cells that constitute vessels had no abnormality without aggregation.

Sural nerve biopsy revealed loss of myelinated and unmyelinated nerves (Fig. 2A and B), a typical finding for CANVAS-related neuropathy^1^. Amyloidosis was excluded by Congo red staining (not shown). An electron microscopic image revealed many collagen pockets (Fig. 2C). Notably, electron microscopic analysis of Schwann cells revealed cytoplasmic inclusion bodies, dense material, or accumulated membranous material (Fig. 2D–G). No apparent abnormality was found in the vascular systems (Fig. 2H).

## Discussion

We found biallelic *RFC1* mutations in three patients with immune-mediated neuropathy and one with non-immune mediated neuropathy. This has been the first study to show an association between repeat expansions in *RFC1* and treatable immune-mediated neuropathy. Patient 1 had typical monophasic GBS with anti-ganglioside antibodies wherein weakness was completely resolved after IVIG, but dysesthesia in the upper limbs remained 3 years after disease onset. Patient 2, who suffered from idiopathic sensory ataxic neuropathy with mild motor deficit, had a recurrent and remission clinical course with some improvement in motor and sensory conditions after repeated IVIG treatment. Patient 3 had MAG demyelinating neuropathy with stable symptoms and decreased IgM levels after rituximab treatment. All three patients tested positive for autoantibodies. Although positivity for rheumatoid factor, IgM anti-IgG, found in patient 2 might not directly cause neuropathy, a previous study demonstrated that rheumatic patients with rheumatoid factor had significantly more frequent neuropathies than those without rheumatoid factor (83% vs. 44%)^14^. Common symptoms in the patients included sensory disturbances, similar to those in Patient 4 with chronic sensory autonomic neuropathy with mild motor deficit and in previous studies on CANVAS^2^.

Pathological studies for CANVAS have been limited, with electron microscopic analyses being reported in only one study^15^. While the reported electron microscopic findings were limited to descriptions regarding Schwann cells associated with unmyelinated axons, we found several rare findings concerning myelinating Schwann cells, with a cytoplasmic inclusion body, dense materials, or accumulated membranous materials. Although Patient 4 did not have electrophysiological evidence of demyelinating neuropathy, subclinical changes in Schwann cells may have occurred. In Patient 2, demyelination was suggested to have occurred in the sural nerve on biopsy at the early stage and in the tibial nerves on NCS at the late stage. In addition, Patient 3 with MAG neuropathy had demyelination in the median nerve on NCS. These findings may indicate that patients with *RFC1* mutations occasionally develop demyelination neuropathy or Schwann cell damage.

A possible mechanism by which *RFC1* mutations are associated with immune-mediated neuropathy includes the vulnerability of the nerves themselves or that of Schwann cells to autoantibodies. In fact, we showed subclinical abnormal findings in Schwann cells producing myelin as mentioned above. Another possible mechanism is the abnormal function of blood–nerve barrier, given that the blood–nerve barrier may protect the nervous system from toxic materials, including autoantibodies^16^. However, we found no abnormalities around blood vessels, the site of the blood–nerve barrier, though the absence of morphological changes cannot rule out its functional change. Alternatively, abnormal immune-response or production of autoantibodies may be promoted in such patients. For example, neuronal or myelin antigens, or an expanded repeat RNA, might be abnormally exposed as immunogens^17^. Despite these fascinating mechanisms, the association between *RFC1* mutations and immune-mediated neuropathy remains inconclusive, because the relatively low incidence of mutations among a large cohort of studied patients may raise the possibility that the association is coincidental.

Biallelic mutations may suggest loss of function in RFC1, which is involved in DNA repair^18^. A recent report describing expansion mutations in an allele and truncation mutations in another supports this mechanism^19^. Loss of DNA repair protein function in neuropathy and cerebellar ataxia is reminiscent of Aprataxin-related disorders^20^. However, a previous study did not find any evidence suggesting loss of DNA repair function of RFC1 in fibroblasts^1^. Our electron microscopic findings suggested cytoplasmic inclusions in Schwann cells, a situation similar to that of SCA2-related neuropathy, a gain of function disease^8^. Further accumulation of evidence is needed to clarify the pathomechanism using neuronal or glial cell models or animal models.

One limitation of this study is the small number of patients with immune-mediated neuropathy who were positive for *RFC1* mutations. In addition, detailed pathological studies in patients with immune-mediated neuropathy were lacking, as mentioned above.

In summary, we found CANVAS-related *RFC1* mutations in patients with treatable immune-mediated neuropathy or demyelinating neuropathy. Thus, immunotherapy should not be terminated solely based on the identification of *RFC1* mutations; however, repeated immunotherapy to unresponsive patients should be avoided^10,21^. Nonetheless, future large-scale studies are needed before definitive conclusions can be established.

### Supplementary Information

Supplementary Table 1.

Supplementary Information.

## Data Availability

Detailed clinical information of patients with *RFC1* mutations were available in the supplemental material.
