# Supplementary material for: CANVAS-related RFC1 mutations in patients with immune-mediated neuropathy
Source: Sci Rep. 2023 Oct 18;13:17801. doi: 10.1038/s41598-023-45011-8 (PMC10584897; doi:10.1038/s41598-023-45011-8)
Supplement: Supplementary file 1 — Supplementary Table 1. [file 41598_2023_45011_MOESM1_ESM.docx]

| Supplemental table 1. Nerve conduction study findings of patients with *RFC1* mutations | | | | | | | | | | | |
| --- | --- | --- | --- | --- | --- | --- | --- | --- | --- | --- | --- |
|  |  |  |  |  |  |  |  |  |  |  |  |
| Patient# | 1 |  | 2 | | |  | 3 | |  | 4 | Normal |
| Main phenotype | GBS |  | ISAN | | |  | MAG neuropathy | |  | SAN |  |
| Note | On admission |  | On admission | Pre IVIG | Post IVIG |  | On admission | Post rituximab |  | On admission |  |
| Antibody | Ganglioside (GQ1b, GT1a, GD1b, and GT1b) |  | RF | | |  | MAG | |  | - |  |
| Repeat configuration | Biallelic AAGGG |  | Biallelic ACAGG | | |  | AAGGG/ ACAGG | |  | Biallelic ACAGG |  |
| Age at onset | 74 |  | 64 | | |  | 56 | |  | 76 |  |
| Age at NCS (yr) | 74 |  | 65 | 75 | 75 |  | 74 | 79 |  | 80 |  |
| Duration of disease* | 3 d |  | 7 m | 10y | 10y |  | 18 y | 23 y |  | 4 y |  |
| Side | Rt |  | Rt | Rt | Rt |  | Rt | Rt |  | Rt |  |
| **Motor Nerve** |  |  |  |  |  |  |  |  |  |  |  |
| *Median Nerve* |  |  |  |  |  |  |  |  |  |  |  |
| DL (ms) | 3.5 |  | 2.9 | 3.2 | 3.3 |  | 6.3 | 5.6 |  | 3.8 | <4.0 |
| CMAP (mV) | 8.9 |  | 10.4 | 8.7 | 8.7 |  | 12.9 | 9.6 |  | 9.8 | >4.0 |
| MCV (m/s) | 52.5 |  | 59.8 | 51.2 | 52.3 |  | 38.0 | 36.5 |  | 52.9 | >54.3 |
| *Ulnar Nerve* |  |  |  |  |  |  |  |  |  |  |  |
| DL (ms) | 3.0 |  | 2.7 | 2.8 | 3.2 |  | 3.5 | 3.8 |  | 2.9 | <3.1 |
| CMAP (mV) | 7.3 |  | 12.5 | 7.1 | 9.4 |  | 7.9 | 8.4 |  | 7.3 | >4.2 |
| MCV (m/s) | 59.0 |  | 58.0 | 54.5 | 48.7 |  | 55.5 | 52.7 |  | 60.3 | >55.5 |
| *Tibial Nerve* |  |  |  |  |  |  |  |  |  |  |  |
| DL (ms) | 5.1 |  | 5.2 | 4.4 | 7.4 |  | 6.8 | 6.0 |  | 5.0 | <5.7 |
| CMAP (mV) | 11.2 |  | 20.4 | 5.8 | 4.9 |  | 9.0 | 8.7 |  | 6.2 | >7.3 |
| MCV (m/s) | 45.1 |  | 47.5 | 36.5 | 38.4 |  | 41.0 | 36.1 |  | 38.8 | >43.9 |
| **Sensory Nerve** |  |  |  |  |  |  |  |  |  |  |  |
| *Median Nerve* |  |  |  |  |  |  |  |  |  |  |  |
| SNAP (μV) | 3.6 |  | NE | NE | NE |  | NE | NE |  | 1.0 | >13.9 |
| SCV (m/s) | 54.7 |  |  |  |  |  |  |  |  | 38.9 | >58.3 |
| *Ulnar Nerve* |  |  |  |  |  |  |  |  |  |  |  |
| SNAP (μV) | 8.0 |  | 15.7** | 1.4 | NE |  | NE | NE |  | NE | >10.8 |
| SCV (m/s) | 41.4 |  | 56.4 | 29.5 |  |  |  |  |  |  | >58.9 |
| *Sural Nerve* |  |  |  |  |  |  |  |  |  |  |  |
| SNAP (μV) | 8.7 |  | NE | NE | NE |  | NE | NE |  | NE | >7.7 |
| SCV (m/s) | 38.7 |  |  |  |  |  |  |  |  |  | >43.5 |
| DL, Distal Latency; CMAP, Compound Muscle Action Potential; MCV, Motor Conduction Velocity; Sensory Nerve Action Potential; SCV, Sensory Action Potential; Abnormal values are underlined; NE, not evoked; nd, not done; *, duration of disease from onset to NCS examination; **, unclear NCS waveform; MAG, Myelin-associated glycoprotein; IVIG, Intravenous immunoglobulin therapy. | | | | | | | | | | | |
