# Supplementary material for: CANVAS-related RFC1 mutations in patients with immune-mediated neuropathy
Source: Sci Rep. 2023 Oct 18;13:17801. doi: 10.1038/s41598-023-45011-8 (PMC10584897; doi:10.1038/s41598-023-45011-8)
Supplement: Supplementary file 2 — Supplementary Information. [file 41598_2023_45011_MOESM2_ESM.docx]

**Supplemental data**

**Primer list for repeat-primed PCR of *RFC1***

RP-FAM-RFC1-F: 5’-[6FAM] TCAAGTGATACTCCAGCTACACCGT-3’

RP-Anchor: 5’-CAGGAAACAGCTATGACC-3’

Plus one of the followings:

For AAGGG (pathogenic)

RP-RFC1-AAGGG: 5’-CAGGAAACAGCTATGACCGGGAAGGGAAGGGAAGGGAA-3’

For ACAGG (pathogenic)

RP-RFC1-ACAGG: 5’-CAGGAAACAGCTATGACCACAGGACAGGACAGGACAGG-3’

For AGGGC (pathogenic)

RP-RFC1-AGGGC:　 5’-CAGGAAACAGCTATGACCGGGCAGGGCAGGGCAGGGCA-3’

For AGAGG (possibly pathogenic)

RP-RFC1-AGAGG:　 5’-CAGGAAACAGCTATGACCGAGGAGAGGAGAGGAGAGGA-3’

For AAGGC (possibly pathogenic)

RP-RFC1-AAGGC:　 5’-CAGGAAACAGCTATGACCGGCAAGGCAAGGCAAGGCAA-3’

For AAAGG (variable penetration)

RP-RFC1-AAAGG: 5’-CAGGAAACAGCTATGACCGGAAAGGAAAGGAAAGGAAA-3’

For AAAAG (normal polymorphism) used with mixture of the below

RP-RFC1-AAAAG1:　5’-CAGGAAACAGCTATGACCAACAGAGCAAGACTCTGTTTCAAAAAAGAAAAGAAAAGAAAAGAAAA-3’

RP-RFC1-AAAAG2: 5’-

CAGGAAACAGCTATGACCAACAGAGCAAGACTCTGTTTCAAAAAGAAAAGAAAAGAAAAGAAAA-3’

RP-RFC1-AAAAG3: 5’-

CAGGAAACAGCTATGACCAACAGAGCAAGACTCTGTTTCAAAAGAAAAGAAAAGAAAAGAAAA-3’

For AAAGGG (normal polymorphism)

RP-RFC1-AAAGGG: 5’-CAGGAAACAGCTATGACCGGGAAAGGGAAAGGGAAAGGGAAA-3’

For AAGAG (normal polymorphism)

RP-RFC1-AAGAG: 5’-　CAGGAAACAGCTATGACCGAGAAGAGAAGAGAAGAGAA-3’

**Patient clinical information**

**Patient 1**

*GBS, biallelic AAGGG repeat expansions*

A 74-year-old woman had fever (38℃) with cough. Ten days later, she started to have an abnormal sensation in her hands. Two days later, she developed an unsteady gait. She then had difficulty raising her arms, opening bottle caps, and moving her jaw. The next day, she had difficulty opening her eyelids and drinking water. She then visited our hospital where she was subsequently admitted. Neurologic examination revealed bilateral ptosis with normal eyeball movement on admission, but disturbed eyeball movement later. Dysarthria and dysphagia were obvious. Muscle weakness was moderate to severe in the upper limbs [manual muscle test (MMT) grade 2–4] and mild in the lower limbs (MMT grade 4–5). Right- and left-hand grip strength was 7 and 10 kg, respectively. The patient complained of dysesthesia in both hands. Although apparent abnormality was noted during light touch, pain sensation was mildly decreased in the right foot. Vibration sensation was decreased in the left foot, but limb ataxia was not apparent. Deep tendon reflexes were decreased or absent in all four limbs. No apparent autonomic nervous system involvement was observed. Vestibular functions were not assessed. She tested positive for anti-ganglioside antibodies: IgG anti-GQ1b, GT1a, GD1b, and GT1b antibodies. Cerebrospinal fluid examination revealed an increase in protein level with a normal cell count. NCS result revealed sensory dominant abnormalities (supplementary table 1). The electrophysiologic diagnosis indicated an unclassified condition on admission according to Ho’s criterion.^14^ However, Hadden's criterion classified her condition under the equivocal category.^15^ Nerve biopsy was not performed. Brain MRI revealed no atrophy in the cerebellum. She was then diagnosed with GBS. After receiving intravenous immunoglobulin therapy (0.4 g/kg/day for 5 days), her muscle strength recovered (MMT 5 levels in all limbs) with normal eyelid elevation and swallowing a month after hospital admission. However, dysesthesia in her upper limbs remained. Three years later (at the age of 77), she still had dysesthesia but no muscle weakness or cranial paraparesis.

**Patient 2**

*Idiopathic sensory ataxic neuropathy with mild motor deficit, biallelic ACAGG repeat expansions*

A 75-year-old woman started to have dysesthesia in her upper limbs at the age of 64 years and in her lower limbs 4 months later. She also developed an unsteady gait and had muscle cramping. She visited our clinic at 65 years of age. After undergoing a check-up in the outpatient clinic, she was admitted to our hospital. Neurologic examination revealed mild weakness in the proximal and distal lower limbs without upper limb weakness. No cranial nerve involvement was noted, with normal eyeball movement. Deep tendon reflexes were retained in all four limbs without pathological reflexes. Sensory deficits were present in both lower limbs across all modalities. No apparent autonomic disturbance was noted. Romberg’s sign was positive. Vestibular functions were not assessed. Cerebrospinal fluid examination revealed a normal cell count and normal protein levels. She underwent biopsy of the left sural nerve, which revealed a decreased number of myelinated and unmyelinated fibers. Teased fiber analysis revealed segmental demyelination in some nerve fibers and short internode lengths in others, suggesting demyelinating neuropathy with remyelination. No vasculitis, atherosclerosis, or inflammatory cell infiltration was found. Unfortunately, although the aforementioned biopsy findings were described in her medical record, pathological materials and images had been discarded after the 10-year retention requirement at the university had elapsed. The patient tested positive for rheumatoid factor, IgM anti-IgG antibody but did not exhibit joint symptoms at that moment. She was a carrier of the hepatis type C virus, which has often associated with positivity for rheumatoid factor. Brain MRI revealed no atrophy in the cerebellum. She was subsequently diagnosed with idiopathic sensory ataxic neuropathy with mild motor deficit and received intravenous immunoglobulin infusion (IVIG, 0.4 g/kg/day for 5 days). Her unsteady gait was resolved with mild amelioration of dysesthesia and disappearance of the Romberg sign. However, she felt worsening of dysesthesia after 4 months and received IVIG. At that time, her Achilles tendon reflexes were mildly decreased. She then developed pain in all four limbs, with wrist deformity and positivity for rheumatoid factor at 66 years of age, suggesting a diagnosis of rheumatoid arthritis. She received medications for rheumatoid arthritis at another hospital but discontinued it due to adverse events (i.e., skin rash), without no detailed medical record present. She tested negative for cryoglobulin. Around 5 months after the second IVIG, she developed an unstable gait and sensory deficits in all four limbs. Deep tendon reflexes in her right upper limb and both lower limbs decreased without pathological reflexes. She received her third IVIG, which improved her gait stability. Around 10 months later, she felt mild weakness and dysesthesia at 67 years of age. Mild weakness was noted in all four limbs. Deep tendon reflexes decreased in her upper limbs and were absent her lower limbs without pathological reflexes. She received her fourth IVIG, which subsequently resolved her weakness. She then developed sensory or mild weakness three times and received IVIG each time. The first conduction block on NCS appeared at 75 years of age as evidence by compound muscle action potentials (CMAPs) of 5.8 and 1.61 mV on distal and proximal site stimulation, respectively, with a motor conduction velocity (MCV) of 36.5 m/s in the right tibial nerve. After her eighth IVIG, her conduction block was resolved, as CMAPs were 4.86 and 4.95 mV on distal and proximal site stimulation, respectively, with an MCV of 38.4 m/s. She refused to take oral prednisolone because of the possibility of adverse events. However, due to small improvements in sensory and motor function, she was lost to follow-up.

**Patient 3**

*MAG neuropathy, compound heterozygous for AAGGG/ACAGG repeat expansions*

A 79-year-old man started to develop dysesthesia in his hands and feet at the age of 56 years. As his dysesthesia progressed, he developed an unsteady gait. After visiting a local hospital, neurologic examination revealed decreased vibration sensation and a positive Romberg sign. Serological analysis revealed increased IgM (470 mg/dL, normal 33–190 mg/dL) but normal soluble IL2-R levels (406 U/ml). As such, he was referred to our hospital at 74 years of age. Neurologic examination at the time revealed no cranial nerve involvement with normal eyeball movement, normal muscle strength, and normal deep tendon reflexes without pathological reflexes. Tandem gait (heel-to-toe walking) disclosed truncal instability. Light touch and pinprick sensation decreased in all four limbs. Vibratory sensation was mildly decreased in his upper limbs and severely decreased in his lower limbs. No apparent autonomic disturbance was noted. Vestibular functions were not assessed. Cerebrospinal fluid examination rivaled a normal cell count and normal protein levels. Serological analysis revealed the presence of monoclonal IgM kappa protein. Moreover, the patient tested positive for IgM-MAG and SGPG antibodies but negative for anti-ganglioside antibody. Electrophysiological analyses revealed possible demyelinating neuropathy, according to the CIDP criteria. No malignancy was suggested by gallium scintigraphy at that time. Brain MRI revealed no atrophy in the cerebellum. His activities of daily living were not significantly altered by his sensory disturbances. Nerve biopsy was not performed. The patient was later discharged. Two years later, prostate cancer (adenocarcinoma) was detected at 76 years of age. Given the presence of iliac lymph node swelling on computed tomography (CT), LH-RH analog treatment was started. Surprisingly, a positron emission tomography (PET) study revealed a hot spot in the palate. Biopsy revealed diffuse large B-cell lymphoma. Hence, rituximab treatment (375 mg/m^2^) was started once a month and decreased to once two month after 6 months. After a year and half, the tumor or lymphoma was no longer detected on magnetic resonance imaging. Rituximab treatment was then terminated. His IgM level subsequently decreased to 268 mg/dL and remained similar (211–285 mg/dL) until the age of 78 years. Dysesthesia and truncal instability were ameliorated given that tandem gait became possible. At 79 years of age, PET-CT detected a hot spot in the left retroperitoneum, with subsequent biopsy revealing recurrence of lymphoma with transformation. Rituximab, pirarubicin, cyclophosphamide, vincristine, and prednisolone treatment was started but was stopped after one course due to adverse effects, such as neutropenia and subsequent aspiration pneumonia. He did not desire further chemotherapy, and was transferred to a hospice.

**Patient 4**

*Idiopathic sensory autonomic neuropathy with mild motor deficit, biallelic ACAGG repeat expansions*

An 80-year-old woman started to develop feelings of dizziness when standing and constipation at 76 years of age. She had a history of olfactory disturbances at 70 years of age. She experienced difficulty with ambulation due to orthostatic dizziness at 79 years of age. She then visited a local hospital. Her orthostatic hypotension was severe from 155/99 mmHg with a heart rate of 85 beats/min to 80/55 mmHg with a heart rate of 70 beats/min. She had decreased or absent deep tendon reflexes with no abnormal plantar responses. No apparent cerebellar ataxia was noted in her limbs, but step-wise horizontal eyeball movement was observed. Truncal ataxia was not evaluated due to severe orthostatic hypotension. She was suspected to have acute sensory and autonomic neuropathy, without serum anti-ganglionic acetylcholine receptor antibody. She received intravenous steroids followed by intravenous immunoglobulins, without any improvement. During the hospital stay, restless leg syndrome was developed. She then visited our hospital where she was later admitted. Neurologic examination revealed muscle wasting in all four limbs and weakness in the peripheral portion of her upper limbs as evidenced by a right- and left-hand grip strength of 4 and 5 kg, respectively. Vibration sensation was severely reduced in her ankles, with relatively preserved superficial sensation. She underwent sural nerve biopsy, which revealed loss of myelination and unmyelinated nerves (Fig 2), a typical finding for CANVAS-related neuropathy.^1^ However, no apparent abnormality was found in the vascular systems. Amyloidosis was excluded by Congo red staining (not shown). Notably, Schwann cells had a cytoplasmic inclusion body or dense materials and accumulated membranous materials. Brain MRI revealed mild atrophy in the cerebrum and cerebellum. She then underwent genetic testing, which showed biallelic ACAGG repeat expansions. Head impulse testing suggested vestibular dysfunction; however, she had been transferred to a local hospital, without detailed vestibular function tests.
